# DeepRNAac4C: a hybrid deep learning framework for RNA N4-acetylcytidine site prediction

**DOI:** 10.3389/fgene.2025.1622899

**Published:** 2025-08-25

**Authors:** Guohua Huang, Runjuan Xiao, Chunying Peng, Jinyun Jiang, Weihong Chen

**Affiliations:** ^1^ Hunan Provincial Key Laboratory of Finance and Economics Big Data Science and Technology, Hunan University of Finance and Economics, Changsha, China; ^2^ College of Information Science and Engineering, Shaoyang University, Shaoyang, China

**Keywords:** convolutional neural network, deep learning, RNA N4-acetylcytidine, BiGRU, BiLSTM

## Abstract

RNA N4-acetylcytidine (ac4C) is a crucial chemical modification involved in various biological processes, influencing RNA properties and functions. Accurate prediction of RNA ac4C sites is essential for understanding the roles of RNA molecules in gene expression and cellular regulation. While existing methods have made progress in ac4C site prediction, they still struggle with limited accuracy and generalization. To address these challenges, we propose *DeepRNAac4C*, a deep learning framework for RNA ac4C sites prediction. *DeepRNAac4C* integrates residual neural networks, convolutional neural networks (CNN), bidirectional long short-term memory networks (BiLSTM), and bidirectional gated recurrent units (BiGRU) to effectively capture both local and global sequence features. We extensively evaluated *DeepRNAac4C* against state-of-the-art methods using 10-fold cross-validation and independent tests. The results show that *DeepRNAac4C* outperforms existing approaches, achieving an accuracy of 0.8410. The proposed *DeepRNAac4C* improves predictive accuracy and model robustness, providing an effective tool for identifying RNA ac4C sites and deepening our understanding of RNA modifications and their functional roles in biological systems.

## 1 Introduction

N4-acetylcytidine (ac4C) is an ancient and evolutionarily conserved RNA modification present across a wide range of organisms, from bacteria to humans (Thalalla Gamage et al., 2021; Zhang Y. et al., 2023; Iqbal et al., 2022). It plays a critical role in various RNA molecules, influencing multiple biological functions and significantly impacting both normal development and disease progression (Karthiya et al., 2020). In both human and yeast mRNA, ac4C enhances translation efficiency and stability by facilitating precise codon recognition (Jin et al., 2020). Additionally, ac4C can also promote gene expression by stabilizing mRNA (Zhang W. et al., 2023). Recent studies have identified potential links between ac4C modifications and cancers such as colorectal and breast cancer, suggesting that ac4C may serve as a promising biomarker for disease diagnosis and therapeutic development (Yang et al., 2021). Therefore, accurately predicting ac4C sites in mRNA is essential for advancing our understanding of RNA translation mechanisms and exploring its implications in disease treatment.

Several approaches have been developed for ac4C site identification, including biochemical and computational methods. Traditional biochemical techniques, such as high-performance liquid chromatography and mass spectrometry, offer precise ac4C detection (Jin et al., 2020). Although these methods provide high accuracy, they are time-consuming, labor-intensive, and require extensive sample preparation. More recently, chemical labeling followed by high-throughput sequencing has emerged as an alternative for transcriptome-wide ac4C profiling. While highly sensitive, this approach may introduce biases due to chemical reactivity and experimental conditions.

In response to these challenges, computational methods, including machine learning (ML) and deep learning (DL), have been widely applied to the field of molecular biology (Huang et al., 2022; Zheng et al., 2023). Several ML-based models have been proposed, leveraging various sequence and structural features. Zhao et al. developed PACES, which integrates a random forest classifier with sequence profiles and nucleotide frequency features (Su et al., 2023). Alam et al. introduced XG-ac4C, an XGBoost-based model for ac4C site identification (Alam et al., 2020). Su et al. proposed iRNA-ac4C, which extracts features from nucleotide composition, chemical properties, and cumulative nucleotide frequency (Su et al., 2023). More recent deep learning models have further improved ac4C site prediction by capturing deeper sequence representations. Lai et al. introduced LSA-ac4C, a hybrid deep neural network combining bidirectional long short-term memory (BiLSTM) and self-attention mechanisms, enhanced by automated machine learning techniques (Lai and Gao, 2023). He et al. proposed NBCR-ac4C, incorporating pre-trained Nucleotide Transformer and DNABERT2 models to construct contextual embeddings and extract multi-level features using convolutional neural networks (CNN) and Residual Network (ResNet) (He et al., 2024). Liu et al. developed TransC-ac4C, which integrates CNN and Transformer architectures to model both local and global dependencies in RNA sequences (Liu et al., 2024).

Despite these advances, several limitations remain. Existing models often struggle to fully capture multi-level hierarchical features from RNA sequences. Many approaches lack effective mechanisms for modeling long-range dependencies, which are crucial for understanding RNA modifications. Furthermore, previous methods may not efficiently integrate subtle feature variations necessary for robust ac4C site prediction. To enhance performance, advanced feature extraction strategies that leverage both local and global sequence representations are needed.

In this study, we propose DeepRNAac4C, a novel deep learning method for ac4C sites prediction. Our model integrates residual neural networks, convolutional neural networks, bidirectional long short-term memory networks, and bidirectional gated recurrent units (BiGRU) to leverage combined features from RNA sequence data. The residual network captures subtle features and residual information from the input data, while the multi-scale CNN extracts sequence features at different scales. The BiLSTM emphasizes the temporal relationships within the RNA sequences, enabling the model to comprehensively understand RNA sequences at various levels. Following feature extraction, a classification module with multiple fully connected layers and activation functions maps high-level features to the final classification output. This design enhances the model’s nonlinear modeling capabilities, making it adaptable to complex relationships. Through this integrated deep learning framework, DeepRNAac4C learns rich features directly from raw RNA sequence data, leading to improved prediction accuracy of ac4C sites. The effectiveness of our method is demonstrated through extensive 10-fold cross-validation and independent testing.

## 2 Materials and methods

### 2.1 Datasets

In this study, we employed the same dataset as iRNA-ac4C (Su et al., 2023), selected for its comprehensive annotation of ac4C sites and demonstrated effectiveness in prior research. In this dataset, the cytidine closest to the ac4C peak was designated as the modification site, with 100 nucleotides flanking each side to form positive samples. Negative samples were randomly selected from non-peak regions, ensuring that all sequences were standardized to 201 nucleotides in length.

The CD-HIT (Li and Godzik, 2006) with a sequence identity threshold of 0.8 was used to filter out highly similar sequences. To maintain dataset balance, an equal number of non-redundant negative samples were selected to match the positive samples. The dataset was partitioned into training and independent testing sets in an 8:2 ratio, resulting in 2,206 positive and 2,206 negative samples for training, and 552 positive and 552 negative samples for independent testing. Table 1 provides a detailed summary of the dataset composition.

**TABLE 1:** The number of positive and negative samples in training and testing sets.

Data type	Training	Testing
Positive	2,206	552
Negative	2,206	552

### 2.2 Methodology

**FIGURE 1:**
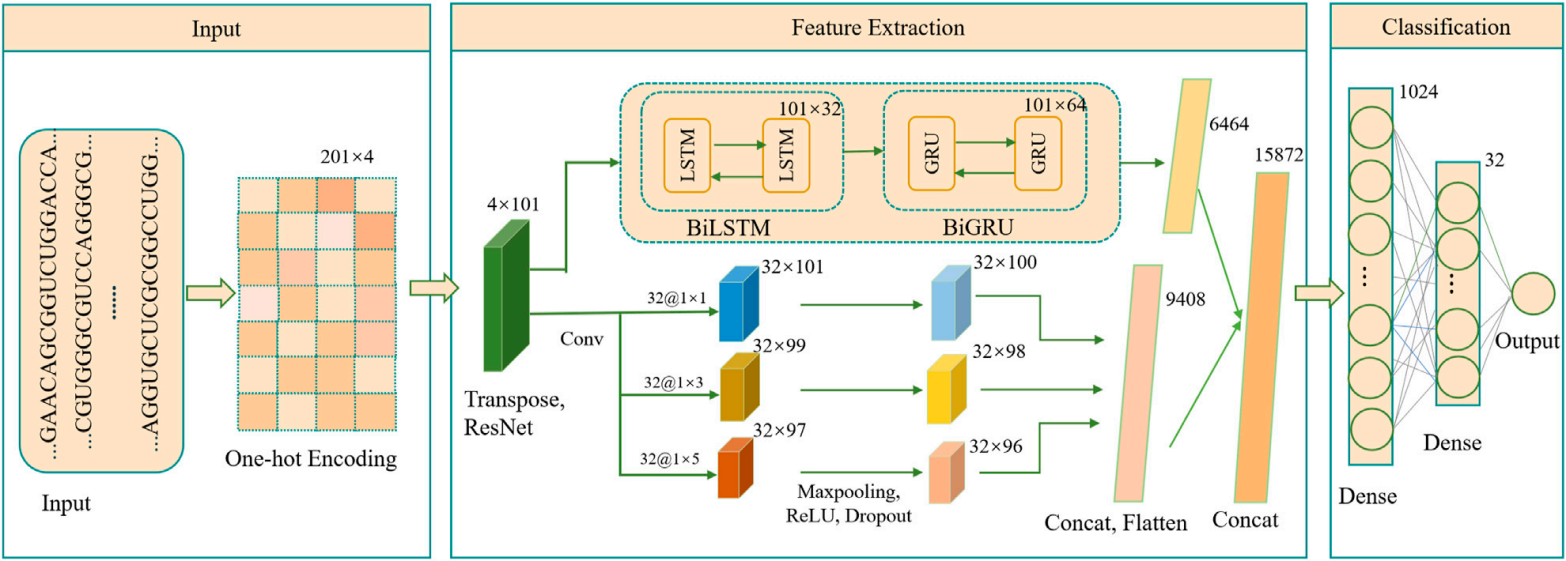
Architecture of the DeepRNAac4C model. *Transpose* refers to matrix transposition, *Cancat* refers to combining multiple inputs, while *Dense* refers to a fully connected layer.

As illustrated in Figure 1, the DeepRNAac4C framework is designed with three key stages: input, feature extraction, and classification. The input stage begins with mapping RNA sequences, each of 201 nucleotides in length, into binary vectors using the one-hot encoding technique. This encoding captures the intricate details of the nucleotide sequences, serving as a robust foundation for the subsequent analysis.

The feature extraction stage employs a combination of residual networks, multi-scale CNN, BiLSTM, and BiGRU. The input data is first passed through a residual network block designed to capture multi-level and abstract features, enhancing the model’s ability to understand complex patterns within the RNA sequences. The output from this block is then processed through a parallel module. One branch of this module incorporates a multi-scale convolutional block, where multiple convolutional filters of different scales are applied in parallel, each followed by a max-pooling layer, ReLU layer and Dropout layer (Wu and Gu, 2015) to improve feature detection and reduce overfitting. This multi-scale approach allows the model to capture features at varying levels of granularity, a crucial innovation for detecting subtle patterns in RNA sequences.

Simultaneously, the other branch of the parallel module utilizes a bidirectional long short-term memory network, followed by a bidirectional gated recurrent unit network. This combination is specifically designed to capture temporal dependencies within the RNA sequence data, thus enhancing the model’s ability to recognize sequence context over time. The outputs from these two advanced modules are then flattened, concatenated, and fed into a well-structured classification module composed of multiple fully connected layers and activation functions. This final stage integrates the rich feature representations obtained from the previous modules to deliver highly accurate classification results, showcasing the power behind the DeepRNAac4C method.

#### 2.2.1 One-hot encoding

One-hot encoding (Agrawal et al., 2022; Karthiga et al., 2021; Zhao et al., 2022) is highly intuitive and easy to understand, as it enables the encoding of biological sequences (such as DNA, RNA, and protein sequences) into binary vectors. For instance, an RNA sequence of length L can be mapped into an L × 4 matrix, where each row represents a base, and each column represents a possible base (A, G, C, U). In this matrix, only the position corresponding to the actual base is set to 1, while other positions are set to 0. Each base has a unique position, and each position has only two possible values (0 or 1). Therefore, one-hot encoding preserves the information of the original sequence. Here, we chose to use a single encoding method rather than combining multiple encoding methods to maintain simplicity and avoid potential complications or noise introduced by combining different encoding strategies.

#### 2.2.2 ResNet

**FIGURE 2:**
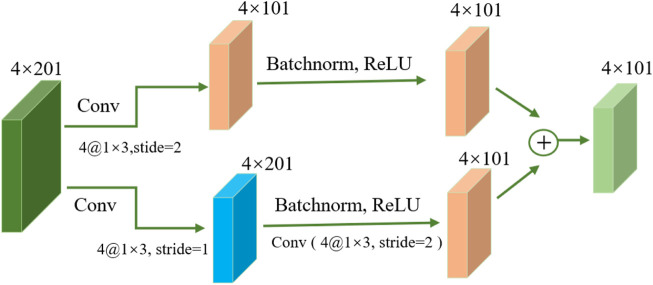
Structure of the ResNet module.

The basic architecture of ResNet (Targ et al., 2016; Wu et al., 2019), illustrated in Figure 2, is built around residual and identity mappings. The residual mapping component comprises convolutional layers, batch normalization layers (Santurkar et al., 2018), Dropout layers, and ReLU activation functions. These layers enhance the network’s ability to extract meaningful features from input data and improve its nonlinear modeling capacity, enabling it to better capture complex data patterns and relationships. Through these layers, the network can better understand complex data patterns and relationships. The key concept of ResNet is residual connections, which allow information to skip connections between network layers. This is crucial for handling very deep networks because, in traditional deep networks, gradients may gradually vanish or explode, leading to training difficulties (Targ et al., 2016). Residual connections enable more stable training by allowing information to bypass some layers, enabling the training of very deep networks (Wu et al., 2019). ResNet ensures that information can flow through the network more effectively without being affected by vanishing or exploding gradients. This improves the efficiency of feature learning, allowing the network to better understand and represent the complex features of input data.

#### 2.2.3 CNN

Convolutional neural networks are pivotal architectures in deep learning, widely applied in various fields such as image recognition (Traore et al., 2018), speech recognition (Passricha and Aggarwal, 2019), and biomedical research (Xu et al., 2021), among others. With the advancement of deep learning, CNNs have become essential components for constructing more complex neural networks. The core characteristic of CNN lies in convolutional operations, enabling the network to learn high-level feature representations of input data, hence CNNs are often referred to as feature extractors.

In CNNs, convolutional layers play a critical role. Neurons in each convolutional layer are connected to a group of adjacent neurons from the previous layer, termed receptive fields. The input and output of convolutional layers are referred to as input feature maps and output feature maps, respectively. Output feature maps represent higher-level representations of input feature maps, generated through sliding convolutional operations with convolutional kernels. To enhance CNN’s nonlinearity, activation functions such as ReLU, sigmoid, tanh, etc., are typically applied to feature maps (Traore et al., 2018). Additionally, pooling layers are utilized for nonlinear down sampling, reducing the dimensionality of feature maps, accelerating computation speed, and helping to prevent overfitting issues.

#### 2.2.4 BiLSTM

Bidirectional Long Short-Term Memory Networks (Passricha and Aggarwal, 2019; Siami-Namini et al., 2019; Tang et al., 2022) are neural network models designed for processing sequential data, particularly adept at capturing contextual information within sequences. In previous Recurrent Neural Networks (RNNs) (Pearlmutter, 1989; DiPietro and Hager, 2020; Yin et al., 2017), although RNNs could handle sequential data and capture contextual information, they faced challenges with long-distance dependencies, such as vanishing or exploding gradients. To address this issue, Long Short-Term Memory Networks (LSTM) (Graves and Graves, 2012; Tsukiyama et al., 2021; Sønderby et al., 2015) were introduced. LSTM replaces some hidden layers of RNN and incorporates a memory mechanism, enabling better capture of long-term dependencies.

Specifically, LSTM selectively adds new information or removes previously accumulated information through the addition of input gates, forget gates, output gates, and candidate cell states, enabling better handling of long sequence dependencies (Yu et al., 2019). The role of the forget gate is to determine how much information from the cell state should be retained at the current time step, thus deciding which previous information should be forgotten. The output of the forget gate ranges from 0 to 1, where 0 indicates complete forgetfulness and 1 indicates full retention. The forget gate is calculated as Equation 1.

$$f_{t}=\sigma \left(W_{f}\cdot \left[h_{t-1},x_{t}\right]+b_{f}\right)$$ (1)

Here, $f_{t}$ is the output of the forget gate, $W_{f}$ and $b_{f}$ are the weight matrix and bias vector of the forget gate, $h_{t-1}$ is the hidden state from the previous time step, $x_{t}$ is the input at the current time step, and σ is the sigmoid activation function.

The input gate controls the input of new information and determines which information to update in the cell state. The output of the input gate also ranges from 0 to 1, determining how much new information to add to the cell state. The input gate is calculated as Equation 2.

$$i_{t}=\sigma \left(W_{i}\cdot \left[h_{t-1},x_{t}\right]+b_{i}\right)$$ (2)

Here, $i_{t}$ is the output of the input gate, $W_{i}$ and $b_{i}$ are the weight matrix and bias vector of the input gate.

The process of updating the cell state involves two key steps: computing the candidate cell state and updating the cell state using the input gate. First, LSTM computes a candidate cell state, which contains the new information to be updated into the cell state. This candidate cell state is obtained by using the tanh activation function to process the linear combination of the input information and the hidden state from the previous time step. This process is computed as Equation 3.

$${\hat{C}}_{t}=\tanh\left(W_{C}\cdot \left[h_{t-1},x_{t}\right]+b_{C}\right)$$ (3)

Where, ${\hat{C}}_{t}$ represents the output of the candidate cell state, $W_{C}$ and $b_{C}$ are the weight matrix and bias vector used to compute the candidate cell state.

Then, LSTM uses the input gate to control whether to add partial information from the candidate cell state to the current cell state, where the output $i_{t}$ of the input gate determines which parts should be updated in the cell state. This is calculated as Equation 4.

$$C_{t}=f_{t}\cdot C_{t-1}+i_{t}\cdot {\hat{C}}_{t}$$ (4)

where $C_{t}$ represents the state at the current time step, and $C_{t-1}$ represents the cell state from the previous time step.

The output gate determines which parts of the current time step’s hidden state and cell state will become the final output. The output of the output gate is a value between 0 and 1, which weights a portion of the cell state using the tanh function. The output gate is calculated as Equation 5.

$$O_{t}=\sigma \left(W_{O}\cdot \left[h_{t-1},x_{t}\right]+b_{O}\right)$$ (5)

where $O_{t}$ is the output of the output gate, $W_{O}$ and $b_{O}$ are the weight matrix and bias vector of the output gate. The hidden state is updated by the output gate and the cell state, which is computed by Equation 6.

$$h_{t}=O_{t}\cdot \tanh\left(C_{t}\right)$$ (6)

To comprehensively capture the semantic information of sequences in sequence analysis, BiLSTM are employed, consisting of two LSTM units: one forward LSTM and one backward LSTM. The forward LSTM learns representations from previous contexts, while the backward LSTM learns representations from the opposite direction. By running two independent LSTM units in both forward and backward directions and concatenating their hidden states, BiLSTM can simultaneously capture both forward and backward information in the sequence. This structure aids in better understanding sequence data, particularly in scenarios involving long-distance dependencies.

#### 2.2.5 BiGRU

BiGRU is an extension of the standard GRU that processes input sequences in both forward and backward directions. GRU, as an improved variant of the traditional RNN, was designed to address the vanishing gradient problem encountered when modeling long sequences (Chuah et al., 2024; Pham NT. et al., 2024). The key features of GRU are summarized as follows.

a) Gating mechanism. GRU introduces two main gates: update gate and state gate. Update gate determines how much previous information needs to be retained in the current state. Reset gate determines how much past information is discarded to incorporate new inputs.
b) State update. GRU can flexibly control the inflow and outflow of information through the computation of Update Gate and Reset Gate, making the model perform better in capturing long-term dependencies.
c) Simplified structure. Compared with LSTM, GRU has a simpler structure and fewer parameters, resulting in faster training and more efficient computation.
d) Widely used. GRU is widely used in tasks such as natural language processing (Gupta and Noliya, 2024; Tawong et al., 2024; Xu et al., 2024), speech recognition (Mehra et al., 2024; Cheng et al., 2024), time series prediction (Zhang et al., 2024), etc. It is especially suitable for scenarios that require capturing sequence context information.

The design of BiGRU gives it better performance and efficiency in processing long sequence data.

#### 2.2.6 Performance evaluation

In this study, we use sensitivity (SN), specificity (SP), accuracy (ACC), and Matthews correlation coefficient (MCC) as evaluation metrics (He et al., 2024; Liu et al., 2024), which are defined as Equations 7-10.

$$SN=\frac{TP}{TP+FN}$$ (7)

$$SP=\frac{TN}{FP+TN}$$ (8)

$$ACC=\frac{TP+TN}{TP+FN+FP+TN}$$ (9)

$$MCC=\frac{TP\times TN-FP\times FN}{\sqrt{\left(TP+FN\right)\left(TP+FP\right)\left(TN+FN\right)\left(TN+FP\right)}}$$ (10)

where TP is the number of true positive samples, TN is the number of true negative samples, FN is the number of false negative samples, and FP is the number of false positive samples. The values of SN, SP, and ACC range from 0 to 1, while the MCC spans from −1 to 1. Larger values of these metrics indicate better performance.

Additionally, we also employed the Receiver Operating Characteristic (ROC) curve as an evaluation metric (He et al., 2024; Liu et al., 2024). The ROC curve is constructed by computing the true positive rate (TPR) and false positive rate (FPR) at various thresholds. FPR is plotted on the x-axis, and TPR is plotted on the y-axis. TPR and FPR are calculated as Equations 11, 12, respectively.

$$TPR=\frac{TP}{TP+FN}$$ (11)

$$FPR=\frac{FP}{FP+TN}$$ (12)

The area under the ROC curve (AUROC) varies from 0 to 1. An AUROC of 1 indicates perfect prediction, 0.5 indicates random prediction, and 0 indicates opposite prediction.

## 3 Results

### 3.1 Performance comparison with various encoding methods

In research, we have chosen to use a single encoding method rather than a combination of multiple strategies to maintain simplicity and interpretability. We evaluated several widely used RNA sequence encoding methods, including Accumulated Nucleotide Frequency (ANF), Basic kmer (Kmer), Adaptive Skip Dinucleotide Composition (ASDC), Dipeptide Binary Encoding (DBE), k-Spaced Nucleic Acid Pairs (CKSNAP), and Nucleotide Chemical Property (NCP) (Chen et al., 2021). These were compared with the one-hot encoding approach to assess their impact on model performance. For feature selection, we utilized traditional support vector machines (SVM) (Chen et al., 2021), leveraging insights from previous studies. SVM is a supervised learning model that constructs an optimal separating hyperplane in a transformed feature space to maximize the margin between distinct classes. The algorithm operates by projecting input features into a higher-dimensional space through kernel functions, then identifying the decision boundary with the largest margin between support vectors. Owing to its margin-maximization property, SVM is particularly effective in high-dimensional data analysis. It inherently assesses feature importance based on the weights assigned in the decision function, thus facilitating the selection of the most informative features.

Table 2 shows the results of the model with different encoding strategies. It is evident that the One-hot + SVM method delivers superior overall performance compared to other encoding strategies, achieving the highest accuracy (ACC: 0.7568) and Matthews correlation coefficient (MCC: 0.5141). These results underscore the strength of One-hot encoding in providing accurate and reliable predictions. While NCP + SVM (ACC: 0.7534, MCC: 0.5072) and CKSNAP + SVM (ACC: 0.7500, MCC: 0.5003) exhibit competitive modeling capabilities, they fall slightly short of the robust performance achieved by One-hot + SVM. This exceptional performance of One-hot encoding can be attributed to its ability to comprehensively represent sequence information, delivering richer and more discriminative features for ac4C site prediction.

**TABLE 2:** Performance comparison with different encoding methods.

Methods	SN	SP	ACC	MCC	AUROC
ANF + SVM	0.6251	0.7385	0.6818	0.3661	0.7575
Kmer + SVM	0.7986	0.3215	0.5599	0.2063	0.6961
ASDC + SVM	0.7584	0.7407	0.7496	0.4993	0.8201
DBE + SVM	0.6750	0.6351	0.6550	0.3105	0.6980
CKSNAP + SVM	0.7534	0.7466	0.7500	0.5003	0.8218
NCP + SVM	0.7625	0.7443	0.7534	0.5072	0.8183
One-hot + SVM	0.7661	0.7475	0.7568	0.5141	0.8201

In terms of sensitivity and specificity, One-hot + SVM demonstrates balanced performance (SN: 0.7661, SP: 0.7475), surpassing methods like Kmer + SVM, which achieves the highest sensitivity (SN: 0.7986) at the cost of extremely low specificity (SP: 0.3215). Additionally, while CKSNAP + SVM achieves the highest AUROC (0.8218), indicative of strong discriminative power, One-hot + SVM and ASDC + SVM follow closely with AUROC scores of 0.8201. These results highlight the consistent and reliable performance of One-hot encoding across all key metrics.

### 3.2 Model selection

#### 3.2.1 Performance comparison with different module combinations

**FIGURE 3:**
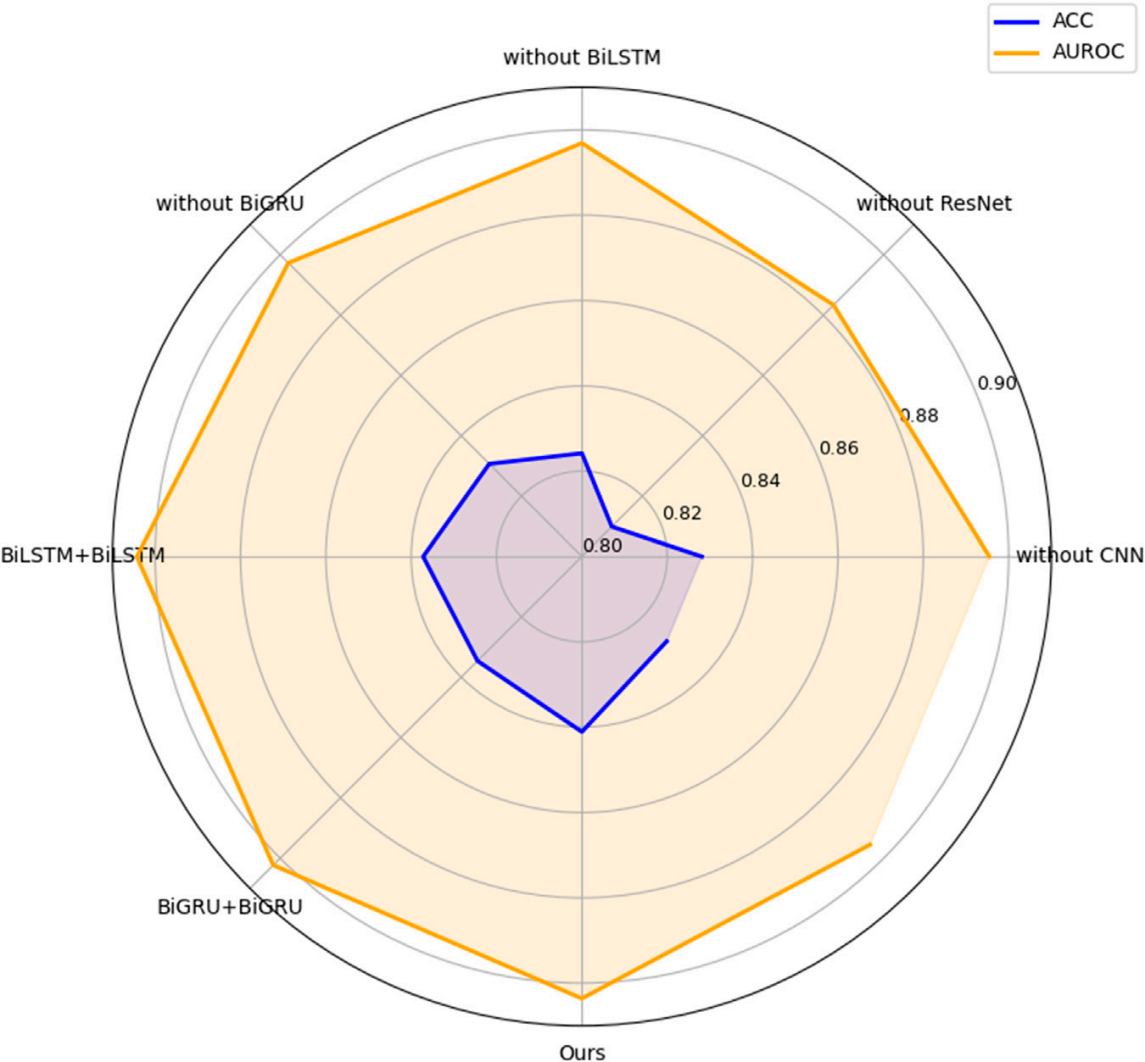
Comparisons of models with different module combinations.

We sequentially compare the performance of models with and without ResNet, BiLSTM, BiGRU, and CNN modules. As shown in Figure 3, the model without the ResNet module exhibited a significant decline in ACC and AUROC, demonstrating its essential contribution to model performance. Accordingly, we retained the ResNet module in the finalized architecture to ensure optimal results. Subsequently, we assessed the impact of the CNN module. The inclusion of CNN led to improvements in ACC and AUROC, reaching 0.8411 and 0.9036, respectively—representing gains of 0.0130 and 0.0202 over the model without CNN.

We further evaluated the contribution of the BiLSTM and BiGRU modules. The model with BiLSTM achieved an ACC improvement of 0.0169 and an AUROC increase of 0.0067 compared to its counterpart without BiLSTM. Similarly, the BiGRU-enhanced model demonstrated gains of 0.0104 in ACC and 0.0063 in AUROC relative to the model without BiGRU. These results highlight the incremental benefits of each module in improving classification performance.

In addition, we assessed the effect of the combination of BiLSTM and BiGRU in the model by testing the combination of BiLSTM + BiLSTM and BiGRU + BiGRU, respectively. BiLSTM captures long-range dependencies by processing sequences in both forward and backward directions, providing rich contextual information. BiGRU, on the other hand, is more computationally efficient and excels at modeling short-to mid-range patterns due to its simplified gating unit. By stacking BiLSTM and BiGRU, the model benefits from a combination of bidirectional processing, deep contextual understanding, and complementary mechanisms, leading to improved ACC and AUROC. The results show that the overall performance of BiLSTM + BiGRU is better than other combinations.

#### 3.2.2 Performance comparison of multi-scale CNN branch configurations

The number of convolutional branches in the multi-scale CNN module significantly influences the model’s performance. In the proposed DeepRNAac4C model, the multi-scale CNN module consists of multiple parallel convolutional branches, each employing a different kernel size (e.g., 1, 3, 5, or 7), thereby enabling the model to capture features across multiple receptive fields. It allows the model to extract both fine-grained and coarse-grained information from the input feature sequence.

As shown in Table 3, increasing the number of branches from 1 to 3 leads to consistent improvements across several performance metrics, including sensitivity, specificity, accuracy, Matthews correlation coefficient, and area under the ROC curve. The configuration with three branches—using kernel sizes 1, 3, and 5—achieves the best overall performance, with an accuracy of 0.8411 and an MCC of 0.6827. This setup effectively balances feature diversity and computational cost, capturing a broad range of informative patterns while avoiding redundancy. In contrast, increasing the number of branches to 4 (with kernel sizes 1, 3, 5, and 7) results in a slight decline in performance. This suggests that additional branches may introduce excessive complexity without corresponding gains in representation power, potentially leading to overfitting or diminished generalization.

**TABLE 3:** The Impact of the number of multi-scale CNN branches on model performance.

Number	SN	SP	ACC	MCC	AUROC
1	0.8929	0.7766	0.8359	0.6751	0.9098
2	0.8801	0.7846	0.8333	0.6685	0.9011
3	0.8699	0.8112	0.8411	0.6827	0.9036
4	0.8724	0.7793	0.8268	0.6553	0.9015

These findings demonstrate that a three-branch multi-scale CNN module offers an optimal trade-off between expressive capacity and model efficiency, making it a preferred choice for robust ac4C site prediction.

### 3.3 Parameter optimization

#### 3.3.1 Performance comparison with different convolutional kernels

In this section, we conducted a comprehensive analysis to evaluate the impact of different multi-scale convolutional kernel combinations on model performance, as summarized in Table 4. Specifically, we tested four configurations: kernel_135, kernel_357, kernel_579, and kernel_157, where the numeric suffix denotes the sizes of convolutional kernels used in the multi-branch structure. For example, kernel_135 refers to the use of three parallel convolutional kernels with sizes 1, 3, and 5; similarly, kernel_357 includes 3, 5, and 7 kernels. Note that 1D-convolution operations are conducted here. These kernel combinations are designed to capture features at varying receptive fields, allowing the model to extract fine-to-coarse details across spatial scales.

**TABLE 4:** Performance comparison of DeepRNAac4C with different convolutional kernels.

Kernel type	SN	SP	MCC	ACC	AUROC
kernel_135	0.8699	0.8112	0.6827	0.8411	0.9036
kernel_357	0.8546	0.8112	0.6667	0.8333	0.9077
kernel_579	0.8878	0.7660	0.6596	0.8281	0.9030
kernel_157	0.8546	0.8085	0.6642	0.8320	0.9067

The results from our evaluation indicate that the model employing kernel_135 achieved the highest performance metrics, with a MCC of 0.6827 and an ACC of 0.8411. Notably, these scores surpassed those of the next best-performing models by margins of 0.016 for MCC and 0.0078 for ACC, highlighting the effectiveness of this particular kernel size.

Furthermore, we observed that the other kernel configurations also demonstrated competitive performance, but none matched the robustness exhibited by kernel_135. For instance, kernel_357, while slightly lower in performance, still provided respectable results with an MCC of 0.6667 and an ACC of 0.8333. Similarly, kernel_579 and kernel_157 had MCC scores of 0.6596 and 0.6642, respectively, illustrating that while they contributed valuable insights, they did not achieve the same level of accuracy as kernel_135.

Based on this comprehensive evaluation, we identified kernel sizes of 1, 3, and 5 as the optimal configuration for our multi-scale convolutional approach. This decision is grounded in the clear performance advantages demonstrated by kernel_135, which we believe will significantly enhance the model’s capability to extract meaningful features from the RNA sequence data, ultimately leading to improved classification accuracy in our DeepRNAac4C method.

#### 3.3.2 Impact of hyperparameter settings

**FIGURE 4:**
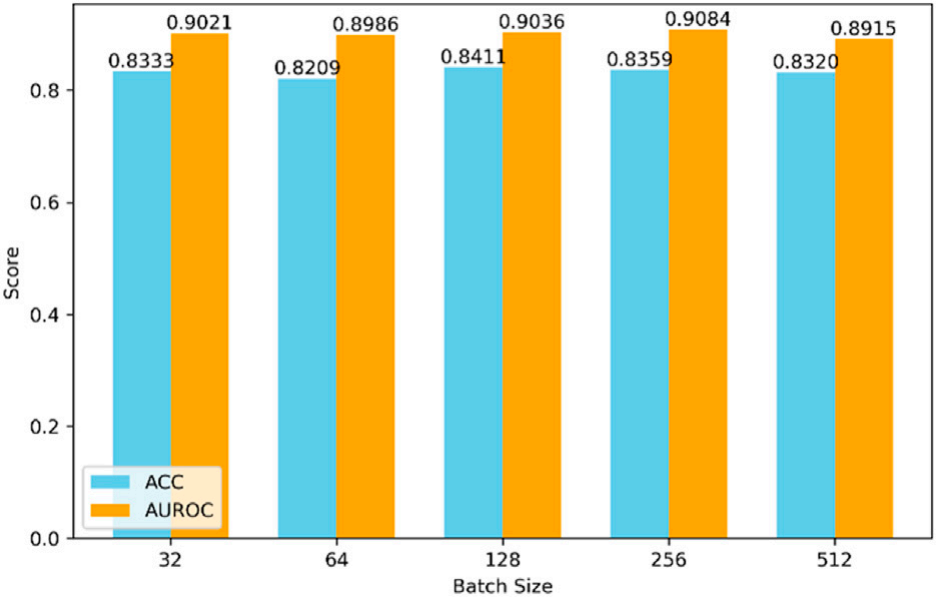
Impact of different batch sizes on model performance.

**FIGURE 5:**
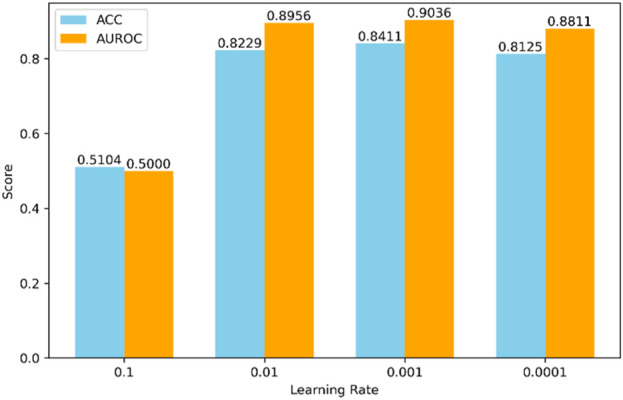
Impact of different learning rates on model performance.

The selection of hyperparameters significantly impacts the final prediction results. Different combinations of hyperparameters may lead to drastically varying performance of the model during both training and testing phases. Due to the nearly limitless range of hyperparameter values and the impracticality of testing all possible combinations, we set some hyperparameters based on experience, as shown in Figures 4, 5.

Firstly, regarding the model’s batch size, as depicted in Figure 4, we compared batch sizes of 32, 64, 128, 256, and 512. Through comprehensive comparison, the model performs best when the batch size is 128.

Secondly, we adjusted the learning rate, a critical hyperparameter, as shown in Figure 5. We tried various values for the learning rate, including 0.1, 0.01, 0.001, and 0.0001. According to the experimental results, the model performs best when the learning rate is set to 0.001.

Finally, based on the above analysis, we selected a batch size of 128 and a learning rate of 0.001 as the final hyperparameter configuration to achieve optimal performance. This choice reflects a thorough evaluation to ensure the model demonstrates outstanding performance during both the training and testing phases.

### 3.4 Performance comparison with state-of-the-art methods

To further validate the proposed DeepRNAac4C, we compared it with existing methods of ac4C site prediction in human mRNA, including PACES (Zhao et al., 2019), XG-ac4C (Alam et al., 2020), iRNA-ac4C (Su et al., 2023), LSA-ac4C (Lai and Gao, 2023), NBCR-ac4C (He et al., 2024), TransC-ac4C (Liu et al., 2024), DPNN-ac4C (Yuan et al., 2024), and ac4C-AFL (Pham NT. et al., 2024). We conducted 10-fold cross-validation and independent testing in our experiments.

In the 10-fold cross-validation, we randomly divided the training set into 10 equally sized or approximately equal parts, with 9 parts used for training and the remaining part used for testing. This process was repeated 10 times. Table 5 presents the performance of the 10-fold cross-validation, with DeepRNAac4C demonstrating well-balanced performance across several key evaluation metrics. Notably, DeepRNAac4C achieves particularly strong results in MCC and AUROC. Although it is slightly less sensitive than XG-ac4C, it shows significant advantages in specificity and overall accuracy, making it well-suitable for a broad range of applications. The results marked with asterisks (*) indicate previously published results, and the values in bold represent the best performance across models. Compared with other methods, DeepRNAac4C excels in all aspects, especially in tasks that require high positive sample detection rates and improved negative sample differentiation. This indicates that DeepRNAac4C provides both high accuracy and stability in predicting ac4C sites in human mRNA.

**TABLE 5:** Performance comparison with state-of-the-art methods based on the 10-fold cross-validation of the training set.

Model	SN	SP	ACC	MCC	AUROC
PACES*	0.7838 ± 0.0186	0.7575 ± 0.0295	0.7706 ± 0.0113	0.5420 ± 0.0219	0.8484 ± 0.0128
XG-ac4C*	**0.9338** ± 0.0123	0.5476 ± 0.0203	0.7407 ± 0.0087	0.5222 ± 0.0162	0.8524 ± 0.0122
iRNA-ac4C*	0.7702	**0.8301**	0.8003	0.6010	0.8750
LSA-ac4C*	0.8554 ± 0.0317	0.7851 ± 0.0313	0.8203 ± 0.0149	0.6431 ± 0.0294	0.8797 ± 0.0118
DeepRNAac4C	0.8717 ± 0.0211	0.7693 ± 0.0347	**0.8281** ± 0.0135	**0.6472** ± 0.0283	**0.8840** ± 0.0187

The “±” symbol represents the mean ± standard deviation, and bold values indicate the best performance. Models marked with an asterisk (*) refer to previously published results [see (Lai and Gao, 2023)]. iRNA-ac4C does not include standard deviation values in the original study.

In independent testing, we trained the model on the training set and evaluated its performance on the test set. As shown in Table 6, the accuracy of DeepRNAac4C in independent testing is comparable to its performance during cross-validation, indicating strong generalization across different datasets, which is essential for real-world applications. Additionally, while DeepRNAac4C may not excel in certain metrics such as SN and SP, a comprehensive evaluation of all five performance metrics highlights its strengths in other critical aspects. Firstly, DeepRNAac4C demonstrates outstanding accuracy with a prediction accuracy of 0.8410, surpassing all current state-of-the-art methods. This signifies the significant capability of DeepRNAac4C in accurately predicting ac4C sites, which is vital for biological research and medical applications. Secondly, DeepRNAac4C’s MCC is particularly impressive, achieving the highest MCC value among existing prediction methods. MCC is widely regarded as a comprehensive evaluation of classification model performance as it simultaneously considers true positives, true negatives, false positives, and false negatives. This indicates DeepRNAac4C’s excellent ability to balance various performance metrics. Lastly, AUROC is a common metric for evaluating binary classification models, and DeepRNAac4C demonstrates high-level performance in this aspect as well. Its AUROC value surpasses all prediction methods, highlighting its outstanding performance across different thresholds.

**TABLE 6:** Performance comparison with state-of-the-art methods on the independent test set.

Model	SN	SP	ACC	MCC	AUROC
PACES	0.7971	0.7790	0.7880	0.5762	0.8648
XG-ac4C	**0.9257**	0.5978	0.7618	0.5542	0.8713
iRNA-ac4C	0.7670	**0.8291**	0.7981	0.5970	0.8800
LSA-ac4C	0.8713	0.7826	0.8270	0.6566	0.8953
NBCR-ac4C	0.8496	0.8207	0.8351	0.6706	0.8958
ac4C-AFL	0.844	0.803	0.823	0.647	0.895
TransC-ac4C	0.8094	0.8045	0.8069	0.6146	0.8691
DPNN-ac4C	0.8178	0.8478	0.8278	0.6578	0.9103
DeepRNAac4C	0.8732	0.8078	**0.8410**	**0.6829**	**0.8971**

The bold values indicate the best performance across models.

In conclusion, DeepRNAac4C excels in its balanced performance across various performance metrics, as well as its outstanding performance in key aspects such as accuracy, MCC, and AUROC. This provides a powerful tool for in-depth exploration of the biological mechanisms of RNA modification and its crucial role in gene expression regulation.

### 3.5 Visualization with UMAP

To further explore the potential and capabilities of our model in distinguishing ac4C sites, we utilized the Uniform Manifold Approximation and Projection (UMAP) (McInnes et al., 2018) technique for visual analysis. UMAP is a powerful method for dimensionality reduction and visualization, enabling us to examine the distribution and clustering of model features in different feature spaces, further explaining the sensitivity and discriminative ability regarding ac4C sites.

**FIGURE 6:**
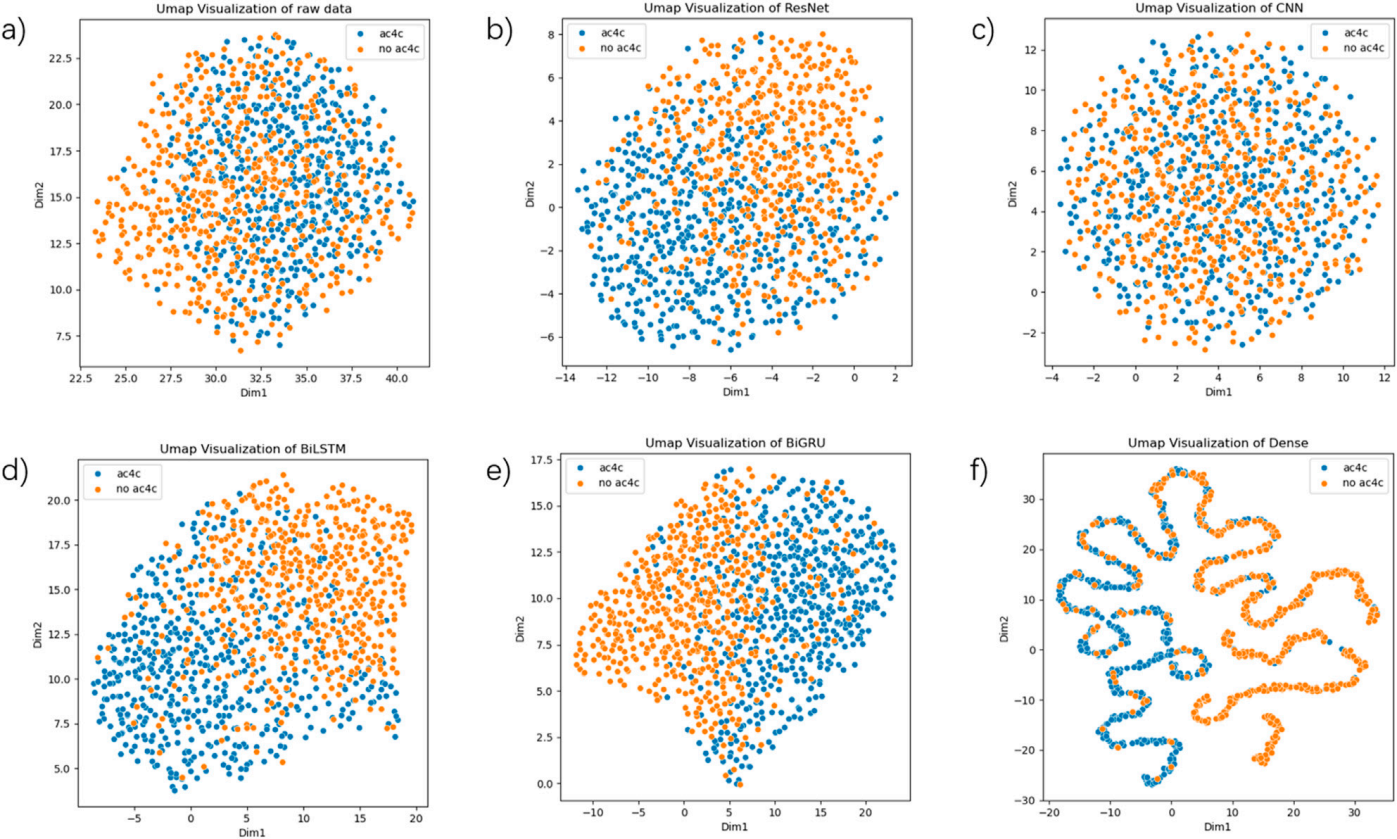
UMAP visualizations based on various layers of the final model. **(a)** depicts a UMAP plot of the raw data. **(b)** depicts the UMAP plot of the output from the ResNet layer. **(c)** depicts the UMAP plot of the output from the CNN layer. **(d)** depicts the UMAP plot of the output from the BiLSTM layer. **(e)** depicts the UMAP plot of the output from the BiGRU layer. **(f)** depicts the UMAP plot of the output from the Dense layer.

As shown in Figure 6, the UMAP plots for different stages of training demonstrate the model’s ability to progressively capture and separate the positive (ac4C) and negative (non-ac4C) samples. In the early stages of training (Figure 6a), the raw data is scattered without clear clustering, indicating that the model is still learning basic features. As the model advances through layers (Figures 6b–e), the feature space becomes more structured, and we see clearer clusters forming (Figures 6b-f). This reflects the model’s ability to learn higher-level, more discriminative features, which aids in distinguishing between ac4C sites and non-ac4C sites. Compared to other methods, the progressive improvement in clustering and sample separation in our model demonstrates the superior discriminative power of our approach. This can be particularly attributed to the hybrid architecture of DeepRNAac4C, which combines multi-scale CNNs and sequential models (BiLSTM + BiGRU). These architectures allow our model to capture both local sequence features and long-range dependencies, providing more informative and distinguishable features than traditional models.

### 3.6 Robustness analysis of DeepRNAac4C

The robustness of DeepRNAac4C is crucial to ensure the method’s stability. In real-world application, training data may contain noise. The method sensitive to noise could not be applied in practice. Therefore, a test for robustness is essential.

In this study, we performed mutation operations (base substitutions) on a portion of positive sample sequences in the training set. The mutation process involved the following rule: randomly selecting 5%, 10%, 15%, and 20% of the positive sample sequences from the training set. Within each selected sequence, we randomly choose an initial position and replace the base at that position and the subsequent bases. We introduced mutations by replacing 10, 20, 30, 40, 50, and 60 consecutive bases, with each base being substituted randomly with one of the four valid bases (A, G, C, U). This process introduced noise into the model training. Subsequently, the model was evaluated on the test set.

**FIGURE 7:**
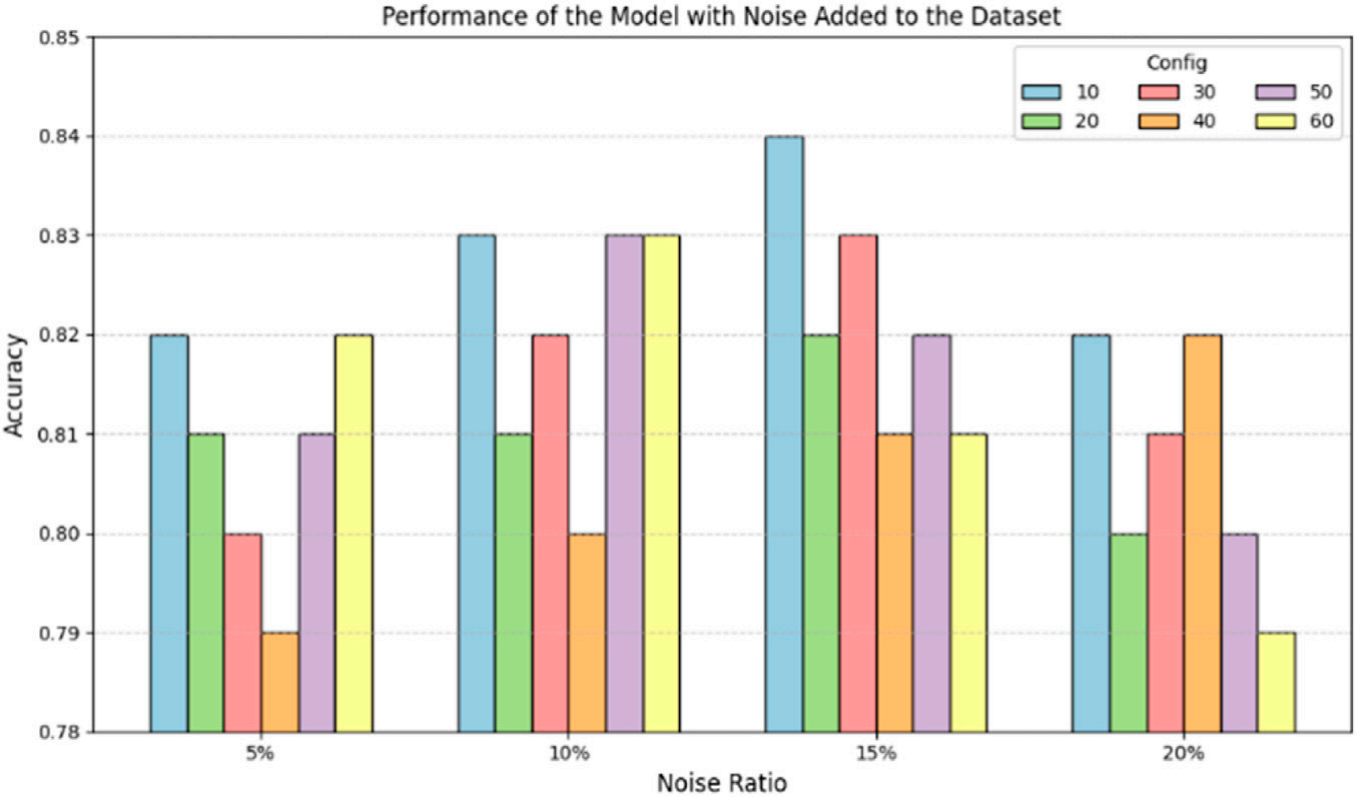
Performance of the DeepRNAac4C model on predicting mutated datasets.

Figure 7 shows the model’s performance after adding noise to the dataset. The horizontal axis represents the number of consecutive base mutations, and the vertical axis represents the model’s prediction accuracy. The different colored bars indicate sequences with varying mutation rates (5%, 10%, 15%, 20%). From Figure 7, it can be observed that after adding noise to the dataset, the model’s prediction accuracy fluctuates between 0.79 and 0.84. Initially, the accuracy decreases slightly with the addition of noise but remains within an acceptable range. This indicates that the model possesses a degree of robustness and can maintain high accuracy even in noisy environments. In addition, the number of consecutive base mutations shows a different trend in terms of their effect on performance. As the number of consecutive base mutations increases, the prediction accuracy of each mutation rate changes differently. For example, when the number of consecutive base mutations is 50–60, the overall performance slightly decreases, but the overall accuracy remains above 0.79.

In conclusion, the DeepRNAac4C model maintains high prediction accuracy even when dealing with noisy, mutated datasets. Although mutation rates and the number of consecutive base mutations can impact the model’s performance, its overall performance remains stable, demonstrating a commendable level of noise resistance and adaptability.

### 3.7 Web server

**FIGURE 8:**
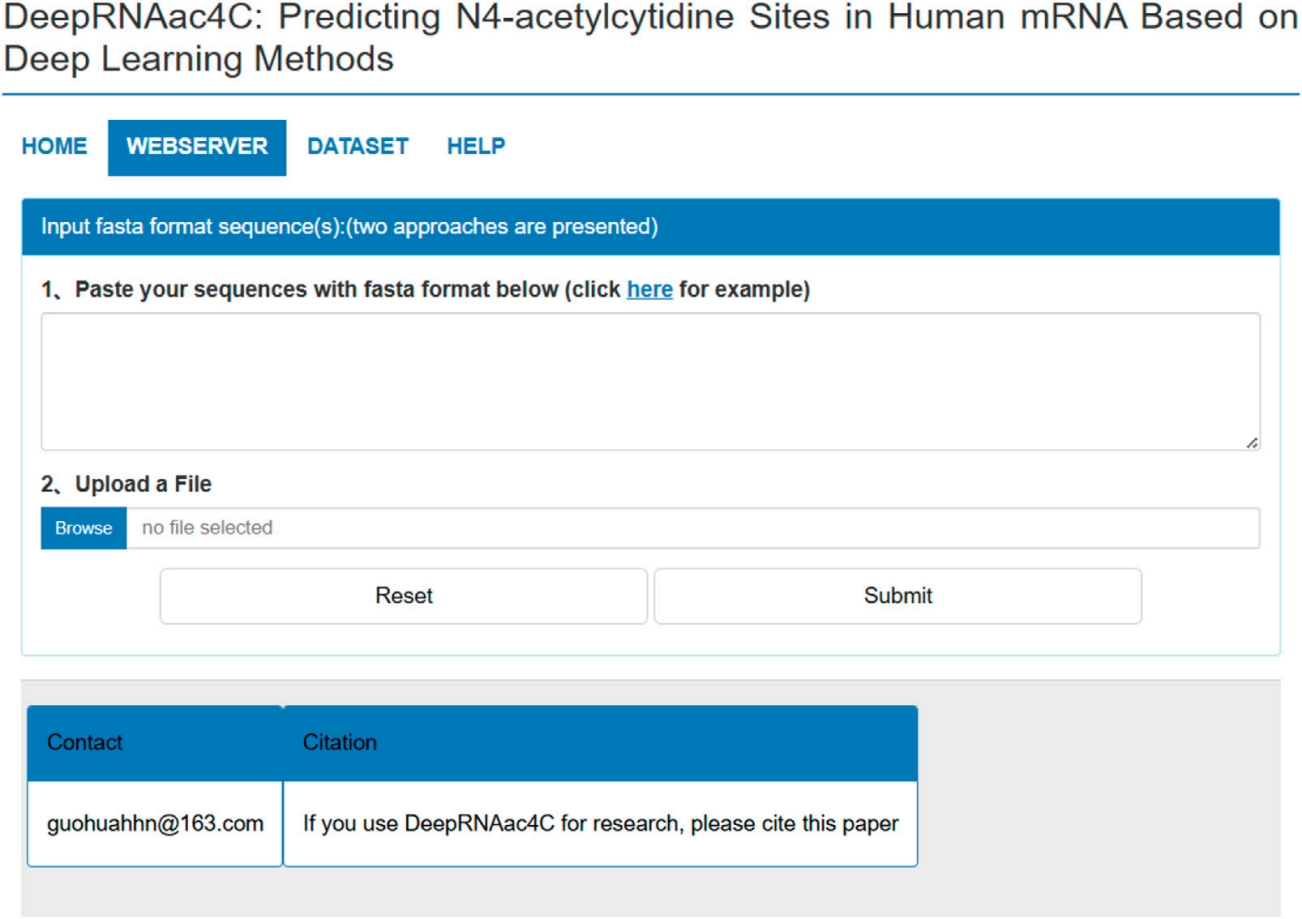
The webserver of DeepRNAac4C.

To facilitate researchers in using the DeepRNAac4C tool more conveniently, we have developed and launched a user-friendly web server, as shown in Figure 8. The DeepRNAac4C server features an intuitive interface and straightforward operation. The following is a brief overview of the usage steps:

a) Submit RNA sequences: Users can submit RNA sequences in FASTA format by either pasting them directly into the input text box or uploading a file. Once the sequences are entered, click the “Submit” button to initiate the prediction process.
b) Wait for prediction results: The server processes the submitted sequences and returns prediction results within a few minutes. The computation time varies depending on the number of sequences submitted, as it is proportional to the input size.
c) Re-submit sequences and dataset download: To re-submit sequences, users can simply click the “Reset” button. Additionally, all experimental datasets can be downloaded by selecting the “Dataset” option on the server.

We believe that the DeepRNAac4C server will be a valuable tool for research on RNA chemical modifications, gene expression regulation, and cell biology, helping scientists make significant advancements in these critical fields.

## 4 Discussion

DeepRNAac4C advances the prediction of N4-acetylcytidine sites in human mRNA by integrating residual neural networks, convolutional neural networks, bidirectional long short-term memory networks, and bidirectional gated recurrent units. This hybrid architecture effectively captures both local and long-range dependencies, overcoming the limitations of previous models that relied on isolated feature extraction mechanisms. The incorporation of residual networks enhances feature extraction by preserving subtle and complex sequence patterns, while multi-scale CNNs enable learning at multiple levels of granularity. Meanwhile, BiLSTM and BiGRU modules strengthen the model’s ability to capture sequential dependencies, improving the prediction of ac4C sites. Experimental evaluations, including 10-fold cross-validation and independent testing, demonstrate that DeepRNAac4C achieves high predictive accuracy, outperforming existing methods. Its high MCC and AUROC indicate a well-balanced performance across positive and negative samples, reinforcing the model’s robustness. Beyond ac4C prediction, the model holds potential for broader applications in biomedical research, particularly in gene regulation, disease mechanisms, and transcriptomics.

Despite the promising results of DeepRNAac4C, several limitations warrant further discussion. First, the current model is trained and evaluated exclusively on human RNA data, raising concerns about its generalizability across species. Given the biological diversity in RNA modification patterns among different organisms, the model’s robustness on non-human datasets remains to be validated. Second, the predictive framework relies solely on primary sequence information, without incorporating RNA secondary structure or chemical modification features, both of which are known to influence the biological functionality of ac4C sites. Third, although the model integrates architectural optimizations such as residual connections and lightweight modules, its hybrid multi-branch design remains computationally intensive. This may limit its scalability in large-scale applications or deployment in resource-constrained environments. Addressing these limitations will require the integration of additional biological modalities, cross-species validation, and further architectural streamlining to improve efficiency without compromising predictive performance.

## 5 Conclusion

This study presents DeepRNAac4C, a deep learning-based approach for accurate ac4C site prediction in human mRNA. By integrating CNNs, residual networks, BiLSTMs, and BiGRUs, DeepRNAac4C effectively captures multi-scale sequence dependencies, addressing key challenges in RNA modification prediction. Extensive evaluations confirm that DeepRNAac4C surpasses existing models, demonstrating high accuracy and robust classification performance. The model provides a valuable tool for advancing research on RNA modifications and their biological significance.

Future work will focus on enhancing model generalizability, integrating RNA secondary structures, and improving computational efficiency to support large-scale transcriptomic analyses. With these advancements, DeepRNAac4C holds promise for broader applications in RNA biology, disease research, and precision medicine.

## Data Availability

The original contributions presented in the study are included in the article/supplementary material, further inquiries can be directed to the corresponding author.
