# Correction: DeepRNAac4C: a hybrid deep learning framework for RNA N4-acetylcytidine site prediction

**DOI:** 10.3389/fgene.2025.1704319

**Published:** 2025-10-13

**Authors:** Guohua Huang, Runjuan Xiao, Chunying Peng, Jinyun Jiang, Weihong Chen

**Affiliations:** ^1^ Hunan Provincial Key Laboratory of Finance and Economics Big Data Science and Technology, Hunan University of Finance and Economics, Changsha, China; ^2^ College of Information Science and Engineering, Shaoyang University, Shaoyang, China

**Keywords:** convolutional neural network, deep learning, RNA N4-acetylcytidine, BiGRU, BiLSTM

To better reflect the broader application of the methods, the phrase has been revised from “applied to predict ac4C sites efficiently” to the more comprehensive “widely applied to the field of molecular biology”.

A correction has been made to the section 1 **Introduction**, Paragraph 3, Line 3: “widely applied to the field of molecular biology”.

To correct an erroneous citation. The reference “Zhao et al., 2019” has been replaced with the correct citation “Su et al., 2023”.

A correction has been made to the section 2 **Materials and methods**, *2.1 Datasets*, Paragraph 1, Line 2: “In this study, we employed the same dataset as iRNA-ac4C (**Su et al., 2023**)”.

For terminological precision and clarity, the sentence “To minimize sequence redundancy, we applied CD-HIT (**Li and Godzik, 2006**) with a similarity threshold of 0.8, filtering out highly similar sequences” is replaced by “The CD-HIT (**Li and Godzik, 2006**) with a sequence identity threshold of 0.8 was used to filter out highly similar sequences”.

A correction has been made to the section 2 **Materials and methods**, *2.1 Datasets*, Paragraph 2, Lines 1–3: “The CD-HIT (**Li and Godzik, 2006**) with a sequence identity threshold of 0.8 was used to filter out highly similar sequences”.

Incorrect capitalization of the term “Convolutional Neural Networks”.

A correction has been made to the section 2 **Materials and methods**, *2.2 Methodology*, 2.2.3 CNN, Paragraph 1, Line 1: “Convolutional neural networks”.

To eliminate redundant wording, the sentence “The calculation formula of the forget gate is calculated as **Equation 1**” is replaced by “The forget gate is calculated as **Equation 1**”.

A correction has been made to the section 2 **Materials and methods**, *2.2 Methodology*, 2.2.4 BiLSTM, Paragraph 2, Lines 9–10: “The forget gate is calculated as **Equation 1**”.

To eliminate redundant wording, the sentence “The calculation formula of the input gate is calculated as **Equation 2**” is replaced by “The input gate is calculated as **Equation 4**”.

A correction has been made to the section 2 **Materials and methods**, *2.2 Methodology*, 2.2.4 BiLSTM, Paragraph 4, Lines 4–5: “The input gate is calculated as **Equation 2**”.

For conciseness, the previously defined term “hyperbolic tangent (tanh)” has been shortened to “tanh”.

A correction has been made to the section 2 **Materials and methods**, *2.2 Methodology*, 2.2.4 BiLSTM, Paragraph 6, Lines 5–6: “This candidate cell state is obtained by using the tanh activation function to process the linear combination of the input information and the hidden state from the previous time step.”

To eliminate redundant wording, the sentence “The calculation formula of the output gate is calculated as **Equation 5**” is replaced by “The output gate is calculated as **Equation 5**”.

A correction has been made to the section 2 **Materials and methods**, *2.2 Methodology*, 2.2.4 BiLSTM, Paragraph 9, Lines 4–5: “The output gate is calculated as **Equation 5**”.

**Equation 6** in [**2 Materials and methods**, *2.2 Methodology*, 2.2.4 BiLSTM, Paragraph 9] was erroneously given as $h_{t}=O_{t}\;\tanh\left(C_{t}\;\right)$ . The correct equation is $h_{t}=O_{t}\cdot \;\tanh\left(C_{t}\;\right)$ .

Terminology has been standardized for consistency. “Onehot” is now correctly spelled as “One-hot”. Additionally, the typo “StratTableegies” has been corrected to “strategies”.

A correction has been made to the section 3 **Results**, *3.1 Performance comparison with various encoding methods*, Paragraph 2, Lines 2–4: “It is evident that the One-hot + SVM method delivers superior overall performance compared to other encoding strategies”.

To remove the redundant and illogical word “both”. A correction has been made to the section 3 **Results**, *3.2 Model selection*, 3.2.1 Performance comparison with different combinations, Paragraph 3, Line 9: “By stacking BiLSTM and BiGRU, the model benefits from a combination of bidirectional processing,”

Incorrect citation in the footnote of **Table 5**. The citation “Zhao et al., 2019” has been corrected to “Lai and Gao, 2023”.

A correction has been made to the section 3 **Results**, *3.4 Performance comparison with state-of-the-art methods*, Paragraph 2, **Table 5** in Page 9: “Models marked with an asterisk (*) refer to previously published results [see (**Lai and Gao, 2023**)].”

To adopt the author-date citation style and provide the complete reference. The numerical citation “**[54]**” has been replaced with “(**McInnes et al., 2018**)”, and the corresponding bibliographic entry has been added to the References section.

One correction has been made to the section 3 **Results** *, 3.5 Visualization with UMAP*, Paragraph 1, Line 3: “the Uniform Manifold Approximation and Projection (UMAP) (**McInnes et al., 2018**)”

The other correction has been made to the section References: “McInnes L., Healy J., Melville J. (2018). Umap: Uniform manifold approximation and projection for dimension reduction. arXiv:180203426.”

Substantive revision to the rationale for robustness validation. The original text “Validating the robustness of DeepRNAac4C is crucial to ensure the model’s generalization capability, which refers to its performance across different datasets and real-world applications. In practical research, data diversity is common, and robustness validation confirms that the model maintains its effectiveness despite such diversity. Additionally, verifying the robustness of the model aids in reproducing research results, ensuring the reproducibility and reliability of scientific findings” has been corrected.

A correction has been made to the section 3 **Results**, *3.6 Robustness analysis of DeepRNAac4C*, Paragraph 1, Lines 1–9: “The robustness of DeepRNAac4C is crucial to ensure the method’s stability. In real-world application, training data may contain noise. The method sensitive to noise could not be applied in practice. Therefore, a test for robustness is essential.”

To accurately report the experimental results, we have corrected the text to match the data in **Figure 7**: 1) The range of accuracy fluctuations has been changed to 0.79–0.84; 2) The lower bound for the overall accuracy has been corrected to above 0.79.

A correction has been made to the section 3 **Results**, *3.6 Robustness analysis of DeepRNAac4C*, Paragraph 3, Lines 7 and 17: “the model’s prediction accuracy fluctuates between 0.79 and 0.84” “the overall performance slightly decreases, but the overall accuracy remains above 0.79”.

To adhere to the standard spelling of the term, the title of Subsection 3.7 is corrected to “Web server”.

A correction has been made to the section 3 Results, 3.7 Web server: “3.7 Web server”.

To eliminate redundant wording, the sentence “The author(s) declare that financial support was received for the research and/or publication of this article.” is to be deleted.

A correction has been made to the section **Funding**, Lines 1–6:

This work was supported in part by the National Natural Science Foundation of China (Grant No. 62272310), and by the Scientific Research Fund of Hunan Provincial Education Department (Grant Nos. 24A0694 and 24A0701).

The original article has been updated.

